# Deep Learning Meets Hyperspectral Image Analysis: A Multidisciplinary Review

**DOI:** 10.3390/jimaging5050052

**Published:** 2019-05-08

**Authors:** Alberto Signoroni, Mattia Savardi, Annalisa Baronio, Sergio Benini

**Affiliations:** Information Engineering Department, University of Brescia, I25123 Brescia, Italy

**Keywords:** deep learning, hyperspectral imaging, neural networks, machine learning, image processing

## Abstract

Modern hyperspectral imaging systems produce huge datasets potentially conveying a great abundance of information; such a resource, however, poses many challenges in the analysis and interpretation of these data. Deep learning approaches certainly offer a great variety of opportunities for solving classical imaging tasks and also for approaching new stimulating problems in the spatial–spectral domain. This is fundamental in the driving sector of Remote Sensing where hyperspectral technology was born and has mostly developed, but it is perhaps even more true in the multitude of current and evolving application sectors that involve these imaging technologies. The present review develops on two fronts: on the one hand, it is aimed at domain professionals who want to have an updated overview on how hyperspectral acquisition techniques can combine with deep learning architectures to solve specific tasks in different application fields. On the other hand, we want to target the machine learning and computer vision experts by giving them a picture of how deep learning technologies are applied to hyperspectral data from a multidisciplinary perspective. The presence of these two viewpoints and the inclusion of application fields other than Remote Sensing are the original contributions of this review, which also highlights some potentialities and critical issues related to the observed development trends.

## 1. Introduction

In the last few decades, hyperspectral imaging (HSI) has gained importance and a central role in many fields of visual data analysis. The concept of spectroscopy combined with imaging was first introduced in the late 1970s in the Remote Sensing (RS) field [1]. Since then HSI has found applications in an increasing number of fields for a variety of specific tasks, and nowadays it is also largely used, other than in RS [2], in biomedicine [3], food quality [4], agriculture [5,6] and cultural heritage [7], among others [8].

Hyperspectral images are able to convey much more spectral information than RGB or other multispectral data: each pixel is in fact a high-dimensional vector typically containing reflectance measurements from hundreds of contiguous narrow band spectral channels (full width at half maximum, FWHM between 2 and 20 nm) covering one or more relatively wide spectral intervals (typically, but not exclusively, in the 400–2500 nm wavelength range) [9]. Current HSI acquisition technologies are able to provide high spectral resolution while guaranteeing enough spatial resolution and data throughput for advanced visual data analysis [10] in a variety of quality demanding application contexts [8].

However, the great richness of HSI come with some data handling issues that, if not correctly addressed, limits its exploitation. The main problem for the computational interpretation of hyperspectral data is the well-known *curse of dimensionality*, related to the great number of channels and to the fact that data distribution becomes sparse and difficult to model as soon as the space dimensionality increases. Nevertheless, the presence of data redundancy (due to the fine spectral resolution and, in some cases, to fairly high spatial resolution) enables the adoption of dimensionality reduction strategies. Doing this while preserving the rich information content is not a simple task, since the spectral–spatial nature of the hyperspectral data is complex, as it can also be observed in terms of inter- and intra-class variability of spectral signatures arising in non-trivial classification problems.

While these difficulties inevitably have repercussions on the performance of traditional machine learning methods, which strongly depend on the quality of (hand-crafted) selected features, relevant solutions to the above issues have been appearing in recent years with the spread of representation learning approaches [11] and their implementation through Deep Learning (DL) architectures.

### 1.1. Hyperspectral Data Analysis Meets Deep Learning

Traditional learning-based approaches to HSI data interpretation rely on the extraction of hand-crafted features on which to hinge a classifier. Starting early on with simple and interpretable low-level features followed by a linear classifier, subsequently both the feature set and the classifiers started becoming more complex. This is the case, for instance, of Scale-Invariant Feature Transform (SIFT) [12], Histogram of Oriented Gradients (HOG) [13] or Local Binary Patterns [14], in conjunction with kernel-based Support Vector Machines (SVM) [15], Random Forests [16] or statistical learning methods [17]. It is interesting to look at the new trend of DL as something whose clues were already embedded in the pathway of Computer Vision and Digital Signal Processing [11,18]. For example, Neural Networks (NN) can approximate what a traditional bag-of-local-features does with convolutional filters [19] very well and SVM can be seen as a single layer NN with a hinge loss. At the same time DL solutions cannot be seen as the ultimate solution for the fundamental questions Computer Vision is called to answer [20].

The advantages introduced with DL solutions lie in the automatic and hierarchical learning process from data itself (or spatial–spectral portions of it) which is able to build a model with increasingly higher semantic layers until a representation suitable to the task at hand (e.g., classification, regression, segmentation, detection, etc.) is reached. Despite these potentials, some attention is needed when DL is applied to hyperspectral data. Most importantly, given the large amount of parameters of DL models (typically of the order of tens of millions), a sufficiently large dataset is needed to avoid overfitting. Hereinafter, large datasets are meant to be composed of hundreds of thousands examples (where a typical example can consist of a spectral signature associated to a pixel or to a small area or a HSI sub-volume). Conversely, a dataset composed of hundreds of examples can be considered small. The very limited availability, where not complete lacking, of public (labeled) datasets is the most evident shortcoming in the current "DL meets HSI" scenario. Due to the curse of dimensionality, the effects of the shortage of labeled training data is amplified by the high data dimensionality and may lead to effects spanning from the so-called Hughes phenomena (classification performance sub-optimalities) to the models’ complete inability to generalize (severe overfitting). Other pitfalls hidden behind limited availability of data for research purposes are limitations in terms of breadth of the studied solutions that may be limited to the scope of the dataset itself. This also leads to the necessity to work with unsupervised algorithms to partially overcome the lack of labeled data. Data augmentation techniques (such as in [21,22]) in conjunction with the use of some specific DL architectures (such as Convolutional Neural Networks and Autoencoders) also play an important role in handling the above data-driven issues.

### 1.2. Purpose and Relations with Other Surveys

The purpose of this survey is to give an overview of the application of DL in the context of hyperspectral data processing and to describe the state-of-the-art in this context. While this review is not meant to gain further insight into technical aspects of specific application fields and instrumentation, its objective is to be at the intersection of these two important trends: DL, driver of disruptive innovation, especially in computer vision and natural language processing, and exploitation of HSI technologies and data analysis, which is expected to have a high growth even beyond the RS field. This two trends meet up in a field where data is at the same time a challenge (for its dimensionality) and a precious resource (for its informative wealth).

**Figure 1:**
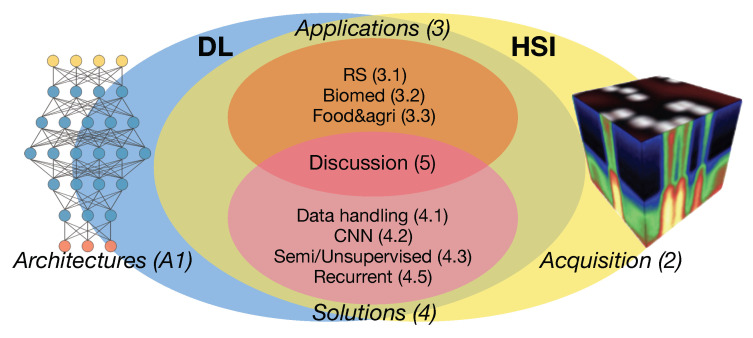
Graphical structure of the article.

Highly informative reviews about DL methods in the RS field have been produced [23,24,25] where there are several references or sections dedicated to HSI data. Conversely, recent work dedicated to reviewing HSI data analysis comprises DL methods [10,26,27,28,29] but their scope is strictly limited to the RS field. With the present work we want to provide an overview of the main principles and advances related to the use of DL in HSI, not only in RS (from airborne or spaceborne platform), but also in other relevant small-scale (from micro to macro ground based acquisitions) applications of HSI data, where DL is already finding fertile terrain for its exploitation. The aim is to define a complete framework to which even non-RS professionals can refer. With this aim in mind, this review has been conceived (and schematized in Figure 1) to be accessible to different categories of readers while maintaining a single and coherent logical flow.

In order to create the context for what follows, in Section 2 we provide a concise overview about the main ways to acquire HSI datasets. This also gives the opportunity to evidence the possibility of exploiting DL solutions in the creation of HSI data from undersampled spectral representations. In Section 3, we adopt the point of view of “what” has been done until now by using DL approaches on HSI data in different application fields. This part is meant to be more accessible to domain expert readers. On the other hand, Machine learning and Computer Vision experts could be more interested in Section 4, which aims to review “how” different DL architectures and their configurations are used on HSI data for different analysis and interpretation tasks. With the final discussion in Section 5, we also want to draw conclusive remarks aimed at pointing out some residual issues and trying to envisage the future developments and challenges to address from the joint exploitation of HSI and DL technologies. Finally, a basic introduction to DL architectures, in particular those mentioned in this work, is provided in Appendix A in order to give additional context and references, especially to domain expert readers.

## 2. HSI Acquisition Systems

In this section we give a concise review of the most diffused approaches that can be exploited for the formation of HSI datasets. Interestingly, we also include a review of recent DL-based solutions conceived for the production of HSI volumes starting from RGB or other sparse spectral representations.

### 2.1. HSI Formation Methods

Hyperspectral imaging (HSI) refers to imaging methods also able to acquire, other than 2D spatial information xy, a densely sampled spectral information λ. The prefix *hyper* is used when the acquired contiguous spectral bands are of the order of $10^{2}$ to $10^{3}$, as opposed to Multispectral imaging (MSI) aimed at the acquisition of order of dozens of bands (with typical FWHM of 100–200 nm), not necessarily contiguous/isometric. Thus, HSI makes it possible to finely capture absorption features, facilitating the identification of the presence of specific substances; while with MSI (and even worse with RGB imaging) physico-chemical absorption features are spread over the channel bandwidth and become much less detectable. Available HSI devices are able to acquire the 3D xyλ volumes by means of 2D sensors ij by converting in time, or arranging in space, the spectral dimension. There are various ways to acquire HSI volumes in practice. Here we review the main and most widespread, each one involving physical limitations requiring a balance between key parameters, such as spectral and spatial resolution, acquisition time (or temporal resolution), device compactness, computational complexity among the main ones.

**Figure 2:**
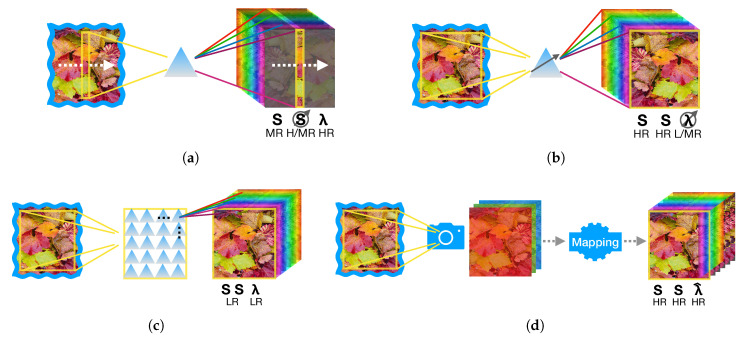
Basic schemes of HSI formation methods. H/M/LR: High/Medium/low Resolution. S: space, either *x* or *y*. λ: spectral dimension. (**a**) Pushbroom linear scanner. (**b**) Spectral selective acquisition. (**c**) Spectrally resolved detector array (snapshot). (**d**) HSI from RGB images.

Relative motion between the HSI sensor and the sample are exploited in whiskbroom (area raster scan) and pushbroom (linear) scanners to respectively acquire the spectrum λ of a single point $x_{i}y_{j}$ (at time $t_{ij}$) or of a line $xy_{j}$ (at time $t_{j}$) of the sample. This is typically done by means of a prism or a diffraction grating able to disperse the incoming light. For whiskbroom mode, the temporal resolution is highly penalized especially if one wants to obtain decent spatial resolution and this prevents, in most cases, the practical use of point-wise spectrography for HSI production. In Figure 2a a pushbroom acquisition is depicted which is far more interesting and widespread since high spatial and spectral resolution can be obtained at the cost of the time needed for the linear scanning over the sample. Commercial pushbroom HSI cameras are currently able to offer easy balancing between frame-rate and spectral resolution (See, for example http://www.specim.fi/fx/ (last visit March 2019)).

Selective spectral acquisition in time is at the basis of another acquisition mode that requires the incoming images to be filtered to produce a $xy\lambda_{k}$ image at time $t_{k}$ (see Figure 2b). The main trade-off here is between spectral and temporal resolution, where spectral filtering can be done with mechanical filter wheels (typically limited to MSI) or by means of acusto-optical or liquid-crystal tunable filters (enabling HSI at a higher cost).

The possibility of obtaining a spectral image by just taking a snapshot is highly attractive for time-constrained applications and this has driven a lot of research [30]. In these cases, physical limitations due to the simultaneous use of spatial and spectral divisions, severely limit both resolutions. Relatively economic systems have been commercialized recently by exploiting a technology able to deposit filter mosaics directly onto the image acquisition chip (See, for example https://www.imec-int.com/en/hyperspectral-imaging (last visit March 2019)). Figure 2c depicts this idea of spectrally resolved detector array, while we refer to [31] for a complete and up-to-date review.

An alternative way to rapidly obtain a HSI dataset from single shots is to derive a pixelwise estimation of $\hat{\lambda }$ by means of an inverse mapping starting from highly subsampled (snapshot) spectral measures, such as RGB images taken by commercial digital cameras. This idea, pioneered in [32,33], has attracted some research interest in the CV community especially toward systems able to simulate the production of HSI images in a very cheap and effective way starting from single RGB images (see Figure 2d). Since in many cases this involved the exploitation of Deep Learning solutions we provide a review of this domain in the next subsection.

### 2.2. HSI from RGB

The possibility to use deep learning approaches to generate hyperspectral images just starting from RGB images, or other sparse spectral representations, has been investigated recently [34,35] and generated a certain interest, especially in the Computer Vision community. The intent is to find alternative solutions to the cost issues and spatial resolution limitations of HSI acquisition devices, by introducing learned inverse mappings from a highly subsampled space to a dense spectral representation.

Different DL solutions (CNN [36,37], 3D CNN [38], Dense and Residual Networks [39], Dirichlet networks [40], Generative Adversarial Networks [41]) have been proposed to improve the mapping and the spectral reconstruction by leveraging spatial context. Following results in [42], which show a non negligible dependency of the spectral reconstruction quality to the colour spectral sensitivity (CSS) functions of the camera, some approaches include the CSS functions to either jointly learn optimal CSS and spectral recovery maps [43], or to produce CSS estimates directly from the RGB images in unknown settings, to better condition the spectral reconstruction [44], or even to learn an optimal filter to construct an optimized multispectral camera for snapshot HSI [45]. A review of recent initiatives in this field can be also found in the report of the first challenge on spectral reconstruction from single RGB images (NITRE 2018 workshop [46]). In a recent work, exploiting properties of computational snapshot multispectral cameras [47], Wang et al. [48] proposed a DL-based HSI volume reconstruction from single 2D compressive images by jointly optimizing the coded aperture pattern and the reconstruction method.

Of course, while these approaches produce interesting results for some applications, their validity is actually limited to the visible spectrum. In fact, to our knowledge no DL-based MSI-to-HSI spectral upsampling has been proposed in the NIR-SWIR spectrum (750–3000 nm) where, because of technological reasons related to currently available detectors, both cost-based and spatial-resolution conditions change and do not lead to the same convenience considerations.

## 3. HSI Applications Meet DL Solutions

In this section we present an overview of DL applications to HSI data subdivided into the main working fields. There is still an imbalance between the number of RS related papers with respect to other application fields. This is due to many factors, including the origins of the development of HSI technologies, the dimension of the RS research community, and the existence of specialized venues. Despite the greater variety and average maturity of works related to RS, in our multidisciplinary review we try to give the greatest value even to exploratory works in other fields being aware that, as it frequently happens, some works done in one domain may inspire other works in another sector.

### 3.1. Remote Sensing

The main purposes of HSI data analysis for RS focus on image processing (comprising calibration and radiometric corrections), feature extraction, classification, target recognition and scene understanding. All these steps are a breeding ground for the exploitation of DL approaches, especially for the potential advantages they bring in terms of data management and feature extraction with a consequent performance boost. Past and future missions (for an updated overview see [49] (Ch. 1)) will feed application professionals with an increasing number of HSI data and big interpretation challenges to address (starting from proper handling of the volume of generated data). Conversely, most of the technological breakthroughs coming from representation learning studies and DL architectures have been quite rapidly tested in RS applications, and RS-related HSI does not represent an exception to this.

#### 3.1.1. Classification

Many DL approaches in the literature include *classification* as a final goal, while land cover classification is one of the main task in RS. The main classes are related to crops (corn, grass, soybean, ...) or urban areas (asphalt, trees, bricks, ...) and, according to available labels in the benchmark datasets, a combination of those classes is considered in the majority of RS-HSI classification works that exploit DL methods.

DL classification architectures have *feature extraction* capability by design. Conversely, classical techniques consider classification on top of a separate hand-crafted feature extraction and remains critical for the representativeness and robustness of the selected features with respect to the task at hand. HSI-DL classification and feature extraction solutions have been recently explored using very different approaches in terms of feature extraction and exploitation. HSI data offer different opportunities to approach the analysis using a pure spectral or a joint spectral–spatial approach. In this section, few works are usually selected as representative of the main paradigms, while in Section 4 many other works are considered according to technological and methodological criteria.Pixel classification can be based on the exploitation of the spectral features thanks to their richness and abundance. Representative studies adopting this approach are [50,51,52,53]. Another kind of classification is based on spatial features, since RS data have a contiguity in space so that classification can exploit the similarities and patterns of neighbouring pixels as in [54,55]. Moreover, jointly considering spectral and spatial features has been proven to enhance the classification, as described for example in [56,57,58,59]. Moreover, the introduction of multiscale spatial features could improve the performance slightly more as demonstrated in [60,61,62]. Yang et al. in [63] tested four DL models ranging from 2D-CNN up to a 3D recurrent CNN model, producing a near-perfect classification result.

Labeled and publicly available HSI datasets (for training and benchmarking) are very few and also quite outdated. The ones considered in the majority of RS land cover classification works are Salinas, Pavia, Indian Pines, and Kennedy Space Center (Information about these datasets can be found at http://www.ehu.eus/ccwintco/index.php/Hyperspectral_Remote_Sensing_Scenes (last visit March 2019)). Moreover, this problem is exacerbated by the current practice in the remote sensing community which carries out training and testing on the same image due to limited available datasets, possibly introducing a bias in the evaluation. Therefore, when this practice is used, this makes fair comparison difficult, since improved accuracy does not always necessarily mean a better approach. As a side effect, this soon leads to accuracy performance that has already compressed and tending to an asymptotic optimal value, and what can generate confusion is that this has happened with very different DL approaches in terms, for example, of number of levels, weights and hyper-parameters to learn. Therefore, even if benchmarking is always valuable, near-perfect results (even obtained taking care of overfitting issues) should not be interpreted as if all land cover classification issues can be considered solved. To reduce the bias deriving from indirect influence of training data on test data when they are taken from the same image (even when random sampling is adopted), a spatially constrained random sampling strategy has been proposed in [64], which can be used in case of limited available labeled HSI volumes.

#### 3.1.2. Segmentation

DL approaches have also been used in RS-HSI for *segmentation* purposes. Hypercube segmentation can be exploited in several ways, and it is useful to better handle a subsequent image classification in several situations. In [65], Alam et al. presented a technique that operates on a superpixel partitioning based on both spectral and spatial properties; in [66] the segmentation of the image was used as a preliminary step to focus the subsequent classification on meaningful and well circumscribed regions.

#### 3.1.3. Target Detection and Anomaly Detection

In RS *target detection and recognition* is receiving increasing interest. In [67,68], parallelized and multiscale approaches were respectively proposed for vehicle detection from satellite images. In [69] Zhang et al. described an oil tank detection system, while in [70] a building detection method was presented.

Target detection could be treated in an unsupervised way as well. In this case, it can be seen, depending on the objective, as *anomaly detection* and usually, it does not need prior information about target objects. These approaches are especially useful, for instance, in the case of forest fire, oil spills in the sea or more in general to detect targets with low probabilities or significant changes that have occurred with respect to a previous acquisition in a certain image scene. Elective areas of application for these methods include, for example, disaster monitoring and defense applications, as well as food processing and various manufacturing related quality controls. Approaches to anomaly detection were found in [71] taking advantage of stacked autoencoders and in [72] where Deep Belief Networks were employed. In [72,73] two different approaches to perform real-time and classical anomaly detection were proposed. Similar to them, in [74], a method exploiting change detection was described. In [75] instead, a DL solution based on Deep Belief Networks and a wavelet texture extraction technology outperformed many baseline models on two HSI datasets.

#### 3.1.4. Data Enhancement: Denoising, Spatial Super-Resolution and Fusion

The physical limitations that characterize the HSI acquisition phase (see Section 2) can relate to issues affecting the quality of the acquired data. This can be partially addressed with data enhancement solutions aimed to increase the practical value or the possibility to exploit the data. A recent example of DL-based solutions in this field is described for *restoration and denoising* in [76], where authors use encoding-decoding architectures as intrinsic image priors to effectively acting as an HSI restoration algorithm with no training needed. With this set-up, they demonstrated the superior capability of 2D priors compared to 3D-convolutional ones, outperforming single-image algorithms and obtaining performance comparable to trained CNNs. A denoising technique powered by CNN is also presented in [77] and related advancements [78,79], where improved noise removal has been obtained with concurrent spectral profile preservation and reduced computational time.

Another popular enhancement task for HSI is (spatial) *super-resolution*. This is aimed to overcome resolution limitations so that, starting from a lower resolved HSI data, high resolution hyperspectral images are produced by exploiting high spatial resolution information coming from another imaging source. This is similar to what happens with *pan-sharpening* [80] where panchromatic images are used to enhance the spatial resolution of satellite MSI data (DL methods have also been applied in this field [81,82]). In general HSI super-resolution comes from the exploitation of RGB or other high-spatial low-spectral images at least in a training phase. To this end, in [83], a simple transfer-learning approach was applied, while in [76,84,85] complete end-to-end architectures were presented. In [86] an end-to-end approach based on 3D convolutions was suggested instead. Within the scope of this work the term end-to-end refers to network architectures that take the HSI volume as input and produce the target data without using separate pre- or post- processing stages. Other approaches are composed of multiple stages in which CNNs are applied extensively as in [87,88] or, more interestingly, without requiring auxiliary images, as in [89].

In certain applications the information provided by HSI alone is not sufficient or, in general, the presence of different and complementary data sources can be exploited to improve results or to enable the accomplishment of specific tasks. This is the case in multi-branch DL solutions conceived to enable *data fusion*, especially involving Lidar and HSI images as in [90,91,92,93,94]. Similarly, in [95] data fusion was carried out on three different data sources, with the inclusion of RGB images as well.

### 3.2. Biomedical Applications

The synergy between HSI and DL can also be exploited in the biomedical sector. For example, the possibility to extract and analyze spectral signatures, spatial maps and joined spatial–spectral representations from specimens in a wide variety of specific application fields (e.g., clinical microbiology, histopathology, dermatology, to name a few) allows the development of (supportive) diagnostic tools in either invasive or non-invasive (or reduced invasiveness) settings. Likewise for RS, where the cover-type classification task is the prominent application, classification operated on the surface of different kinds of specimens, acquired through HSI systems at various scales (from micro to macro), is gaining high interest [3]. Concurrently, the adoption of DL solutions is rapidly becoming the first choice when approaching the majority of medical image analysis tasks [96]. However, despite the high potential, the number of studies able to fully take advantage of both HSI and DL technologies is still relatively low. This may be due to the fact that HSI acquisitions in many biomedical fields are still experimental and unconventional, other than leading to a high amount of data that may be difficult to handle. There are also cost factors and other experimental challenges in terms of infrastructure and experimental setup that, despite the conceptual non-invasiveness of HSI acquisitions, still interfere with a wider usage of HSI systems. However, the interest in HSI and modern DL-based handling of the produced data can grow towards well integrated, safe and effective investigation procedures, and the emerging studies we examine below are proof of this.

#### 3.2.1. Tissue Imaging

The discrimination between normal and abnormal conditions was pursued in an exploratory study [97] to assess the presence of corneal epithelium diseases by means of CNN. In [98,99] different 2D-CNN solutions were considered to classify head and neck cancer from surgical resections and animal models, respectively. Other studies further investigated the possibility of delineating tumor margins on excised tissues [58] and to demonstrate a richer “optical biopsy” classification of normal tissue areas into sub-categories like epithelium, muscle, mucosa [100], also by means of deeper CNN architectures and full spatial–spectral patches. In an interesting study, where a dual-mode endoscopic probe was developed for both 3D reconstruction and hyperspectral acquisitions [101], a CNN-based system was proposed to obtain super-resolved HSI data from dense RGB images and sparse HSI snapshot acquisitions. The latter were obtained by exploiting linear unbundling of a circular optical fiber bundle.

#### 3.2.2. Histology

The task of cell classification is another conceptually similar discrimination that was explored in [102,103] to recognize white blood cells in microscopy images, where different bands were acquired by exploiting Liquid Crystal Tunable Filters (LCTFs). Conversely, in [104], an two-channel global-local feature end-to-end architecture was proposed for blood cell segmentation and classification. Increased spectral information at pixel level can also be exploited as a sample-preserving alternative to invasive chemical procedures, such as in [105], where a virtual staining network was tested to possibly avoid chemical staining of histopathological samples.

#### 3.2.3. Digital Microbiology

In the field of clinical microbiology, multi-class classifications, based on CNN and softmax output, were used for the recognition of bacteria species over VNIR (visible near-infrared, 400–1400 nm) HSI acquisitions of bacteria culture plates where spectral signatures was extracted from single bacterial colonies [106,107]. Interestingly, the exploitation of spectral signatures at a colony level can be seen as an alternative to another form of chemical staining taking place when so called chromogenic culturing plates (filled with agar media enriched with species-selective pigmentation agents) are used to introduce some colour differentiation among bacteria species. This is also significant in recent years since clinical microbiology laboratories are interested by an epochal change in terms of automation and digitization of the whole culturing processes [108]. As a side issue of possible massive usage of HSI data one should consider data conservation needs, typically arising in biomedical domains, which can lead to data handling (storage and transfer) problems especially for high spatial–spectral resolution HSI volumes, each one typically occupying hundreds of MB in raw format. Therefore studying adequate compression techniques and strategies capable of guaranteeing the preservation of the classification and discrimination performance is of high interest, especially in contexts characterized by a high data throughput, such as digital microbiology, where bacteria culturing is massively performed for routine exams and a great volume of digital data is created on a daily basis [109].

#### 3.2.4. Vibrational Spectroscopic Imaging

Despite our focus on HSI, it is worth observing that, especially in the biomedical field, vibrational spectral imaging techniques [110,111] have also recently started to benefit from the possibility offered by representation learning approaches to directly analyze raw spectra (avoiding pre-processing and/or manual-tuning), even improving performance with respect to more classical machine learning solutions [112]. In [113], automatic differentiation of normal and cancerous lung tissues was obtained by a deep CNN model operating on coherent anti-Stokes Raman scattering (CARS) images [114]. In the context of histological applications of Fourier Transform Infrared (FTIR) spectroscopic imaging [115], CNN-based approaches have been introduced to leverage both spatial and spectral information for the classification of cellular constituents [116] and to accomplish cellular-level digital staining to the micrometer scale [117].

### 3.3. Food and Agriculture

HSI techniques are widely recognized for their added value in the agricultural field for a variety of monitoring, modeling, quantification and analysis tasks [6], while in the food industry sector, noninvasive and nondestructive food quality testing can be carried out on the production and distribution chain by means of HSI-based inspection [118]. Examples of HSI-DL techniques were used to assess the freshness of shrimps [119,120] and to prevent meat adulteration [121]. In agriculture either pre- or post-harvesting controls can be conducted. In the first case nutrient inspection [122] or early pathogenic diagnosis [123] were tested, while the possibility of post-harvesting controls were investigated with the assessment of fruit ripening indicators [124], to help segregate damaged fruits [125] and to detect the presence of plant diseases [126]. The main rationale of adopting DL-based data analysis and interpretation combined with HSI is the need to fully exploit the richness of spectral (frequently linked to chemometric principles in the NIR range) and spatial (usually related to the complexity and non-uniformity of the samples) information, contrasting the complexity of hand-crafted feature extraction by relying on representation learning and DL abstraction hierarchies. Additional complexity can also derive from environmental variables that interfere in case of acquisition in the open field, as in [123]. Discrimination among different (plant) species is another salient application that was trialled in the case of cereal [127] or flower [128] varieties.

### 3.4. Other Applications

HSI-DL works in other application fields are still very rare. The authors of a recent review of HSI applications [8] proposed a solution for ink analysis based on CNN for automated forgery detection [129] in hyperspectral document analysis [130]. Interesting developments can be expected within the scope of historical and artistic document analysis (manuscripts, paintings, archaeological artifacts and sites), forensic analysis, anti-counterfeiting and authentication domains, surveillance and homeland security, to name a few.

## 4. Deep Learning Approaches to HSI

In recent years, a variety of DL approaches and architectures have been proposed to address the HSI analysis task described in the previous section. We will mainly focus on Convolutional Neural Networks (CNN) in different configurations (spectral, spatial, spectral–spatial) which have primarily been employed with the aim of feature extraction and classification. In doing so, we will introduce various methods, from classical networks to the integration with multiscale and fusion strategies, as in [131]. Other significant architectures we consider are Autoencoders, Deep Belief Networks, Generative Adversarial Networks and Recurrent Neural networks (all concisely revised in Appendix A). These architectures are flexible and adaptable to different data analysis tasks and suit HSI analysis as well. Dataset augmentation, post-processing solutions and an overview about new directions in HSI data handling conclude this section.

### 4.1. Data Handling

Hyperspectral data can be treated according to different spatial–spectral viewpoints. Most of the early DL methods only exploit data pixel-wise (1-dimensional approaches), working in the spectral direction. This can be done by extracting spectral signatures from single pixels or from groups of them either surrounding a central pixel or belonging to an object area. The latter approach generally needs some a-priori knowledge and a pre-processing phase to detect the object of interest (by segmentation). In [107] a spectral *cosine distance transform* is exploited to identify and weight pixels belonging to objects of interest in a biomedical application.

Dimensionality reduction is used to tackle the spectral information redundancy. Of the different dimensionality reduction techniques, PCA is still a classic way to proceed. Depending on the context, other approaches can be used as well, such as ICA [132] and stacked autoencoders [66].

Otherwise, a 2-dimensional process can be applied. In this case a preliminary dimensionality reduction is usually carried out as well. Spatial processing is exploited to extract spatial features from the whole bands or on 2D patches.

Finally, HSI data can be handled as a whole with the aim of extracting both spatial and spectral features (3-dimensional). Some of these approaches still use a pre-processing stage to condition the data, but often the final goal is to work directly on the "raw" hypercubes. Since this can be a computationally expensive and complex way to proceed, operating on 3D patches (i.e., sub-volumes) is often a preferred method.

### 4.2. Convolutional Neural Networks

Nowadays CNNs are the most popular DL approach in computer vision, thanks to their ability to include additional meaningful restriction in the learning process, like space-invariant features and robustness to slight rotation and deformation. They can also work with a limited training dataset thanks to new and powerful regularization techniques, which are one of the most important characteristics behind their success. In the following subsections we first consider CNNs when they are mainly used as feature extractors (Section 4.2.1). We then map the remaining CNN-based approaches according to whether they work with only one (spectral or spatial) data characteristic (Section 4.2.2) or if they jointly exploit the spectral–spatial nature of HSI data (Section 4.2.3). Where not otherwise specified, classification objectives are related to pixel labeling according to the land cover classes defined in the benchmark datasets (see Section 3.1.1). In Table 1 the HSI-DL papers reviewed in the current section are subdivided into their application domain categories.

#### 4.2.1. Cnn as a Feature Extractor

CNNs have often been combined with classical ML methods, especially SVM. In this setup a CNN is used as a way to dynamically learn a feature extractor from data. This approach has the advantage of exploiting the ability to automatically retrieve a good feature set, from the CNN side, and the robustness to overfitting even on small datasets, from the classical machine learning side. In [136] Leng et al. described a hybrid CNN-SVM for hyperspectral land-cover classification, in which a target pixel and the spectral information of its neighbours are organized into a spectral–spatial multi-feature cube without extra modification of the CNN. In [97] a CNN was combined with SVM to perform binary classification (injured vs healthy) on a small ophthalmic dataset. In [67,68], the introduction of a multiscale approach has proved to be important for the extraction of robust features.

More complex architectures were proposed to jointly handle the space and spectral dimensions in order to produce a more complete feature representation. For instance, in [138] a two-channel deep CNN was used to produce spectral–spatial features from hyperspectral images for land cover classification. Wei et al. [137] proposed a hierarchical framework called spectral–spatial Response that jointly learns spectral and spatial features directly from the images.

In order to perform a robust feature extraction which squeezes all information within HSI data, many methods proposed to optimize and join spatial and spectral features in a single setup. The *fusion* may also involve features extracted from multiple sources and at different levels to make full use of HSI and, for instance, Lidar images as in [91,92,94]. Similarly, in [90] Chen et al. proposed a method in which spatial and spectral features are extracted through CNNs from HSI and Lidar images respectively, and then are fused together by means of a fully connected network. Instead, Xu et al. [95] presented a pixel-wise classification method based on a simple two-channel CNN and multi-source feature extraction. In particular, a 2-D CNN is used to focus on spatial feature extraction and a 1-D CNN is used for spectral features. Eventually, a cascade network is used to combine features at different levels from different sources (HSI, Lidar, RGB). In [134] a two-stream CNN was trained with two separate streams that process the PolSAR and hyperspectral data in parallel before fusing them in a final convolutional layer for land cover classification. A recent effort in this field has been made in [135], in which Jiao et al. proposed a framework for hyperspectral image classification that uses a fully-convolutional network based on VGG-16 to predict spatial features starting from multiscale local information and to fuse them with spectral features through a weighted method. Classification is then carried out with a classical method (SVM). A similar approach was taken in [133] with the addition of a new objective function that explicitly embeds a regularization term into SVM training.

#### 4.2.2. Spectral or Spatial Approaches

Supervised 1D-CNN working at pixel level was proposed in different domains [50,123,128,139] to directly exploit the information relative to each spectral signature. This usually leads to better results with respect to classical ML approaches. For instance in [140], authors proposed an ad-hoc model, carefully tuned to avoid overfitting, providing better results with respect to a comprehensive set of shallow methods. However, especially in the RS domain, performance of pixel-wise methods can be affected by noise [50]. To cope with noise, averaged spectra can be extracted by a group of pixels belonging to an object of interest. This approach is particularly suitable in small-scale domains as in the case of segmented rice seeds [127]. In [107], a similar approach was used in a biomedical scenario, where signatures were obtained by a cosine distance weighted average of pixels belonging to segmented bacterial colonies.

Principal Component Analysis (PCA) is a technique highly exploited in RS to handle data dimensionality and it is used to pre-process data in many DL pipelines as well. In [102], CNN classification of pixel patches obtained after PCA reduction was proposed for cell classification. PCA was used also in [103] to pre-process medical HSI data and improved performance was obtained by the combination or modulation of CNN kernels with Gabor kernels in the preliminary network layers, as suggested in [165].

A different approach for spatial feature extraction was presented by Zhao et al. in [54], and its evolution in [61], in which a multiscale CNN was introduced to learn spatial features. With respect to other methods, data are reorganized into a pyramidal structure containing spatial information at multiple scales. In [55], a band selection method based on spatial features was proposed in order to maximize the HSI classification under the small training set constraint. Similarly, in [141], band selection was performed by means of a distance density measure. The produced spectral signature was then fed to a CNN trained on full bands, exploiting the advantage of a rectified linear unit (only activated for non-zero values), in order to test the band combinations without retraining the model.

#### 4.2.3. Spectral–spatial Approaches

Working jointly with both spectral and spatial features generally leads to improved results. In [163], Zhang et al. described a dual-stream CNN that exploits spectral features using a method similar to [50], spatial features with the approach presented in [139], and a softmax regression classifier to combine them. A similar dual-input approach exploiting a concatenation of spectral and spatial features extracted with 1D-CNN and 3D-CNN respectively was proposed in [121], in a food adulteration detection context. A three-dimensional CNN-based approach can be exploited to extract combined features directly from the hyperspectral images to be used in classification, as done in [126] for plant disease identification. In [157], this allowed to obtain important results in the RS domain, also thanks to a combined $L_{2}$ regularization to avoid overfitting and the use of sparse constraints. A similar approach was also described in [144,147] where spectral–spatial feature extraction and consequent classification were done directly on hypercubes and without any pre-processing. The work in [146] presented a similar approach, but with a Siamese CNN [166].

In [58,100], Halicek et al. proposed an effective 3-D CNN based on AlexNet, trained with 3-D patches and an extended version with an inception block (i.e., with filters of multiple sizes operating at the same network level). While in [164], Gao et al. introduced a network with an alternate small convolution and a feature reuse module able to improve the rate of the high-dimensional features in the network, thus allowing a better extraction. In the last few years, RS-HSI research has been particularly focused on this kind of architectures. Densenet-like architectures and VGG16 were also exploited in [135,156], respectively, for classification. In [158], Liu et al. described a 3-D CNN trained via deep few-shot learning [167] to learn a metric space that causes the samples of the same class to be close to each other. This approach has proven to be effective in cases of few labeled data.

An interesting improvement to a CNN-based model was introduced by Paoletti et al. in [150], where the redundant information present in hidden layers was used in order to exploit additional connections between them in an efficient way, generally enhancing the learning process. One additional 3-D approach was proposed in [159] and recently in [160]. In the latter case a complex scheme was proposed, in which virtual sample creation and transfer-learning were adopted in order to mitigate data shortage during training.

Other examples of spatial–spectral approaches were found in [148,153], in which CNN pixel classification methods that hierarchically construct high level features were presented. Furthermore, in [145] a sparse representation method was employed to reduce the computational cost and to increase the inter-class discrimination after the feature extraction from CNN while, in [155], this step was followed by a spectral feature reduction method. In [151] an architecture that extracts band specific spectral–spatial features and performs land cover classification was presented. Yang et al. [152] used a two stream spatial–spectral network to perform transfer-learning, by fine-tuning only final layers, producing an improvement with respect to excluding the transfer-learning part. In [143] Lee et al. first tried to use a very deep CNN, proposing a Contextual Deep CNN for classification, which is able to jointly optimize the spectral and spatial information together.

A multiscale-based approach is presented in [154], in which multiscale object features, obtained from an initial SLIC (simple linear iterative clustering) superpixel segmentation [168], were combined with spectral features and used as input to a CNN for classification. Instead, in [57] authors proposed a Diverse-region-based CNN (DR-CNN), which uses a joint representation from diverse regions in the proposed CNN framework, simultaneously taking advantage of both spectral and spatial features. Furthermore, they adopted a multiscale summation module designed to combine multiple scales and different level features from unequal layers.

In [161], Ouyang et al. demonstrated that networks augmented by reconstruction pathways can bring some advantages to feature extraction and classification. The reconstruction is established by the decoding channel with reconstruction loss computation, which is then used jointly with the classification loss as the loss function for network training. Finally, the high-level features from the encoding and decoding channels are combined by a designed control gate. This is somewhat similar to what can be achieved with the deconvolutional network used in [162] aimed at recovering images starting from the intermediate features in order to improve the training.

The introduction of sensor-specific feature learning (a model is trained to learn the separability of a sensor using a specific dataset) leads to architectures able to produce good feature sets for classification purposes. In [149] Mei et al. created a sensor-specific five layer structure integrating both spatial and spectral features. Fang et al. [142] proposed a new architecture that is capable of adaptively selecting meaningful maps for classification produced by a multi-bias module that decouples input patches into multiple response maps.

Recently in [62], 1D, 2D, and 3D multiscale approaches were compared with a new multiscale- convolutional layer, demonstrating the superiority of the proposed 3D approach.

### 4.3. Autoencoders and Deep Belief Networks

Autoencoders (AEs) and Stacked Autoencoders (SAEs) have been widely used in hyperspectral imagery for different tasks, mainly in RS but also in food-quality applications. This is due, as in Deep Belief Networks (DBN), to the fact that they tackle the problem of small labeled datasets by attempting to exploit an unsupervised or semi-supervised approach before the desired training, thus producing a well initialized architecture that is suited to HSI tasks.

In [59] this approach was used and tested on RS-HSI for the first time by Lin et al. They proposed a framework in which PCA on spectral components is combined with SAEs on the other two dimensions to extract spectral–spatial features for classification. In line with this in [169] Chen et al. presented different architectures where spectral, spatial (flattened to 1-D vector by using PCA) or jointly driven classifications are obtained by a Logistic Regression (LR) layer operating on features computed with SAEs. Similarly, in [170,171] a SAE was used, followed respectively by a SVM and a Multi Layer Perceptron (MLP) for the classification. In the food quality domain, SAE-based approaches were used in combination with regression methods to predict and quantify the presence of chemical indicators of food freshness [119,120,122] or to assess edible quality attributes [124]. In [172], Ma et al. proposed an effective method called Contextual Deep Learning (CDL) which can extract spectral–spatial features directly from HSI. In order to exploit spectral feature extraction in [52] Karalas et al. used sparse AE composed of a single hidden layer, as well as stacked in a greedy layer-wise fashion; in [66] the same goal was reached using a segmented SAE by employing a dimensionality reduction.

An improvement to plain SAE was introduced by Ma et al. [173] in order to deal with parameter instability when a small training set was used. In particular a SAE is modified not only to minimize the classification error as usual, but also to minimize the discrepancy within each class and maximize the difference between different classes. In [174] an improved version with deep SAE was presented. Zhang et al. [71] proposed a stacked autoencoder suitable for hyperspectral anomaly detection.

Multiscale approaches were also introduced to support AE. In [54] Zhao et al. proposed a combination of AEs and LR. In particular they introduced a method that combines PCA to extract spectral features, multiscale convolutional AEs to extract high-level features and LR to classify them. In [175] a mixture between SAEs and CNN was used. In particular SAEs are exploited to generate deep spectral features (1-D) which are then combined with spatial features extracted with a pyramid pool-based CNN able to manage features at different scales. On top of it, a LR classifier is used.

Many works use stacked denoising AEs, which are SAEs trained on noisy input. Liu et al. [176] used them to generate feature maps that are then classified trough a superpixel segmentation approach and majority voting. In [53], Xing et al. presented a pre-trained network using stacked denoising AEs joined with a logistic regression to perform supervised classification. Conversely, in [82] modified sparse denoising AEs were used to train a mapping between low-resolution and high-resolution image patches for pan-sharpening. Inspired by denoising AEs, an unsupervised DL framework, namely Relit Spectral Angle-Stacked AE (RSA-SAE), was employed in [177] to map hyperspectral image pixels to low-dimensional illumination invariant encodings. In Ball et al. [178], a complete classification pipeline was presented, in which a denoising SAE is fed using an augmentation technique, and a final post-processing provides robust image classification. Lan et al. [179] proposed a framework integrating k-sparse denoising AEs and spectral–restricted spatial characteristics for hyperspectral image classification.

Thanks to their dimensionality reduction capabilities DBN can be used to extract features. In [180] DBN were combined with LR classification, similarly to how SAEs were exploited in [169]. In [56] 1-layer-DBN and 2-layer-DBN with spatial–spectral information were both used after a preliminary PCA. Recently, an unsupervised DBN was presented in [72] by Ma et al. to develop a real-time anomaly detection system able to detect interesting local objects. Instead, in [75], DBNs were fed with a 3D discrete wavelet transformation on the input HSI data. Autoencoders also find applications in non-linear spectral unmixing, for endmember extraction and abundance map estimation. In [181] a solution that relies on the given data and does not require supervision is presented, while in [182] an end-to-end learning method called EndNet is introduced based on an AE network exploiting additional layers and a Spectral Angle Distance metric.

### 4.4. Generative Adversarial Networks

Generative Adversarial Networks (GANs) have gained a lot of interest for their ability to learn to generate samples from data distribution using two competing neural networks, namely a generator and a discriminator. In [183], authors used the discriminator network of a trained GAN to perform classification. This method has proven to be effective when the number of training examples is small. Similarly, [184,185,186] applied GANs in order to use their discriminator outputs for the final classification phase. In [105] a conditional generative adversarial network (cGAN) was used to build a mapping from PCA reduced HSI data and RGB images of chemically stained tissue samples.

### 4.5. Recurrent Neural Networks

Other DL approaches worth mentioning are those based on *Recurrent Neural Networks* (RNNs), i.e., neural network architectures specifically designed to handle time dependencies. In this case, hyperspectral data can be treated as if they were video sequences (with spectral bands as video frames) and a RNN can be applied to model the dependencies between different spectral bands, as in [187]. In [51], Mou et al. presented a supervised classification method which focuses on the use of RNN and parametric rectified *tanh* as an activation function. In [146] Liu et al. introduced a bidirectional-convolutional long short term memory (LSTM) network in which a convolution operator across the spatial domain is incorporated into the network to extract the spatial feature, and a bidirectional recurrent connection is proposed to exploit the spectral information. Recently, Shi et al. [188] presented a 3-D RNN able to address the problem of the mixed spectral pixel in order to remove the noise in the classification stage.

### 4.6. Dataset Augmentation, Transfer-Learning, and Unsupervised Pre-Training

A way to address the lack of availability of labeled pixels is by using different data augmentation strategies. Among them, random pixel-pair features (PPF) was introduced in [21], which exploits the similarity of the pixels of the same class to augment the training data, where a deep CNN with multi layers is then employed to learn these PPF. This approach was improved in [22], in which Ran et al. proposed a spatial pixel-pair feature, SPFF, with a flexible multi-stream CNN-based classification. In [189] Windrim et al. proposed a data augmentation strategy based on relighting for training samples of the CNN which consists of simulating the spectral appearance of a region under different illumination during training. While in [190], Li et al. made an extensive comparison of common augmentation techniques and proposed a new one that helps the CNN to better learn intra-class correspondences.

Another way to handle this data availability problem is to exploit big labeled datasets containing similar data with a *transfer-learning* approach. The reasoning is that usually the first part of a DNN learns generic filters that are reusable in many contexts. In [191], Windrim et al. used this approach by creating a pre-trained CNN on a similar but more complete HSI dataset and then fine-tuning it on the ground-truth dataset. The advantage is that the ground-truth dataset can be now considerably smaller and the training procedure faster. Similarly a transfer-learning approach was employed in [73] to build an anomaly detection system that works on the difference between pixel pairs or in [192] for classification on both homogeneous and heterogeneous HSI data.

As mentioned above, the lack of training sets makes unsupervised and semi-supervised methods increasingly interesting. For example, in [193], Ratle et al. proposed a semi-supervised neural network framework for large scale HSI classification. In [194], Romero et al. presented a layer-wise unsupervised pre-training for CNN, which leads to both performance gains and improved computational efficiency. In [195], Maggiori et al. introduced an end-to-end framework for dense pixel-wise classification with a new initialization method for the CNN. During initialization, a large amount of possibly inaccurate reference data was used, then a refinement step on a small amount of accurately labeled data was performed. In [196], Mou et al. proposed, for the first time in HSI, an end-to-end 2-D fully Convolution-Deconvolution network for unsupervised spectral–spatial feature learning. It is composed of a convolutional sub-network to reduce the dimensionality, and a deconvolutional sub-network to reconstruct the input data.

Advanced training strategies that use semi-supervised schemes were also presented. These made use of abundant unlabeled data, associating pseudo labels in order to work with a limited labeled dataset as in [197], where a deep convolutional recurrent neural networks (CRNN) for hyperspectral image classification was described. Instead, in [93], a ResNets architecture capable of learning from the unlabeled data was presented. It makes use of the complementary cues of the spectral–spatial features to produce a good HSI classification.

### 4.7. Post-Processing

Conditional Random Fields (CRF) have been used in several works thanks to their ability to refine CNN results for different tasks. In [65], Alam et al. presented a technique that combines CNN and CRF operating on a superpixel partitioning based on both spectral and spatial properties, while in [198], CNNs were combined with Restricted CRF (CNN-RCRF) to perform high-resolution classification, refining the superpixel image into a pixel-based result. Recently, in [199], a decision method based on fuzzy membership rules applied to single-object CNN classification was adopted to increase classification performance with a considerable gain in accuracy.

### 4.8. New Directions

Finally, we consider other recent solutions that manage HSI data in a more sophisticated way or that can be considered interesting directions deserving further investigation.

*Training sample restrictions* Specific DL models and training methods have been proposed to improve accuracy when the number of training samples is not abundant. In [200], the inherent spatial–spectral properties of HSI data were exploited to drive the construction of the network model. The use of an edge preserving filter allows us to better explore the contextual structure in a resilient way with respect to noise and small details. An extension of this approach has been proposed in [201] with the introduction of a multi-grain and semi-supervised approach. A self-improving CNN was described in [202] that is able to handle data dimensionality and the lack of training samples by iteratively selecting the most informative bands. In [203] a domain adaptation method was used to exploit the discriminative information of a source image to a neural network for HSI classification.

Active transfer learning is an iterative procedure of selecting the most informative examples from a subset of unlabeled samples and can be used to train deep networks efficiently [204] also with small training sets. Active learning was used in [205] in order to search for salient samples and is able to exploit high-level feature correlations on both training and target domains. Instead, Haut et al. [206] performed spectral–spatial classification using Active Learning coupled with a Bayesan-CNN, where the idea was to add a prior distribution, allowing a probability or likelihood to be defined on the output.

*HSI enhancement* As discussed in Section 3.1.4, many sources of degradation negatively impinge on the overall quality of HSI. Thus, different solutions has been proposed in order to recover a high-quality HSI both in the spectral and spatial dimensions. In the area of super-resolution, it is worth mentioning the work by Yuan et al. [83] in which a transfer-learning procedure was applied, and the method in [207] that combined both spectral and spatial constraints with a CNN model. Conversely, in [84], a super-resolution network was employed to improve a classification module in an end-to-end fashion. Remarkably, this approach only used a small amount of training data. Instead, Lin et al. [101] proposed a new architecture called SSRNet (super-spectral-resolution network) that is able to estimate dense hypercubes from standard endoscope RGB images and sparse hyperspectral signals from a RGB to HSI base reconstruction and a sparse to dense HSI refinement. Similarly, an image recovery CNN from spectrally undersampled projections was proposed in [35]. Another HSI super-resolution method [208] took inspiration from deep laplacian pyramid networks (LPN). The spatial resolution is enhanced by an LPN and then refined, taking into account the spectral characteristics between the low- and high-resolution with a non-negative dictionary learning. In [79] Xie et al. presented a promising quality enhancement method. It combines the theory of structure tensors with a deep convolutional neural network (CNN) to solve an HSI quality enhancement problem.

*Capsule Networks* A new kind of approach in the computer vision field that is currently growing is *Capsule Neural Network*. This kind of network has the aim of improving the CNN robustness to geometric transformations using Capsules, a nested set of neural layers that provide the model with a greater ability to generalize. Examples are found in [209,210,211,212]. In particular, in [210], Wang et al. proposed a 2-D CapsNet for HSI classification by using both spatial and spectral information, while in [212] Yin et al. introduced a CapsNet architecture with pretraining and initialization stages to improve speed and convergence while avoiding overfitting.

*Classification related tradeoffs* In real systems, other requirements/limitations, e.g., in terms of data occupancy or power consumption, can conflict with (classification) performance maximization. The high data flow imposed by HSI in quality inspection or high throughput diagnostic procedures is a challenge when mid- or long-term data conservation is a requirement: for example in [109] authors evaluated the combined use of classification and lossy data compression. To this end, after selecting a suitable wavelet-based compression technology, they tested coding strength-driven operating points, looking for configurations likely able to prevent any classification performance degradation. The result showed that it is possible to derive guidelines for using lossy compression to concurrently guarantee the preservation of the classification quality and the highest compression rate. When computational complexity or power consumption restrictions do emerge, it becomes relevant to evaluate classification performance trade-offs with respect to model implementations on low-power consumption architectures [213]. Concerning computational speed, in [214], Paoletti et al. proposed an implementation of 3-D CNN by integrating a mirroring strategy to effectively process the border areas of the image.

## 5. Discussion and Future Perspectives

**Figure 3:**
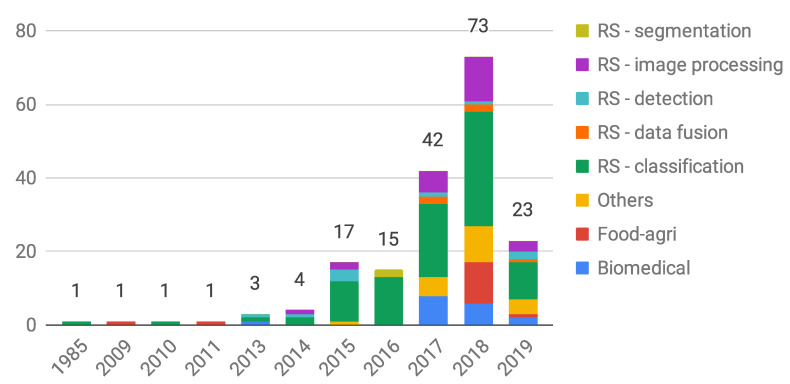
Number of HSI-DL articles per year. The last column comprises published and in-press papers found up to 31 January 2019.

An imbalance that clearly emerged from this overview is the one between the number of HSI-DL studies in the scope of RS with respect to the ones in other application fields. This is depicted in more detail in Figure 3 where, on an annual basis, we subdivided HSI-DL works in this survey by application areas, with RS related studies further split into sub-fields. In this count we did our best to include literature works and their subject mapping. In case of large overlaps of content in multiple works only the most representative works were included. The aforementioned disparity derives from multiple factors: historical and technological reasons (hyperspectral imaging started and developed first and foremost in the RS sector); the development of a wide scientific community; the existence of many venues (journals and conferences) dedicated to RS research themes.

Almost all HSI-DL RS scientific papers, however, still refer to a limited amount of publicly available datasets. While this has proved to be a powerful enabling factor and a stimulus for relevant technological advancements and benchmarking, it can be associated to the risk of incremental and self-referential scientific production as well. Therefore, despite an apparent abundance and exponentially increasing trend (see Figure 3) in the number of RS-related studies (especially for land cover classification), there is still considerable scope and need for the development of workable supervised and unsupervised (or semi-supervised) HSI-DL solutions dedicated to classification studies in specific sub-fields (like soil and geology, water resources and environmental studies, agriculture and vegetation, urban and land development, etc.) as well as vast potential to work on other relevant tasks like change, target, and anomaly detection, analysis of data coming from different sensors (data fusion), spectral unmixing and physico-chemical parameter estimation. Moreover, segmentation is a path not yet well traveled. Architectures like U-net or V-net (for a volumetric approach) can be a good choice to start with, but its formulation in this scenario is yet to be investigated. There is a large variety of HSI classification problems requiring a tailored design or an accurate assessment of existing DL solutions. To comply with the specific application requirements, complexity and computational issues as well as hardware optimization must enter the selection of suitable approaches in addition to pursuing satisfactory accuracy performance. Unfortunately, however, the limited amount of available data also involves difficulties in comparing different methods that lead to similar results, and this again happens for RS image classification studies on benchmark datasets, where near perfect results have been obtained by several, more or less complex, architectures [27,29]. Additional generalization problems arise for data characterized by a relatively high intra-class spectral–spatial variability, not only due to intrinsic target variability but also to atmospheric and daylight conditions. To mitigate these issues, specific data augmentation techniques deserve further investigation, where new generative networks based on GANs can produce very good synthetic data and new and more powerful augmentation techniques. Reinforcement learning could play an interesting role in the near feature in this field as well.

With due proportion, the development in non-RS applications fields seems to be following an important increasing trend as well. This could be the beginning of a successful era in the field of HSI data analysis characterized by a virtuous circle of new industry and professional usages and the development of new acquisition and lighting devices. The market penetration of these systems needs to be backed up by equipment cost reductions, commitment to the generation of representative datasets, the development of advanced DL-based data analysis solutions, and the exploitation of optimized HW/SW computational platforms. This scenario could lead to favourable cost-benefit evaluations and to a greater diffusion of HSI-DL technologies in industrial and professional fields. This could eventually lead to a desirable expansion of dedicated research communities as well. For example, since HSI analysis is still relatively new in many fields related to Computer Vision, there shall be great potential in the future for further investigations in this area from different perspectives, such as 3D modelling and reconstruction, object detection, motion and tracking, multi-sensor data analysis and fusion, etc.

In the professional and industrial fields, datasets are acquired with a precise application purpose and the parameters of the acquisition setup can normally be well controlled by favouring the design of ad-hoc solutions. Although small-scale HSI scenarios can present a high variability, the collection of data is sometimes facilitated as well as the commitment to find resources for data labeling or metadata production by factors such as the race to product development or the mandatory nature of the diagnostic processes. In case of over-abundant availability of data this can be exploited with unsupervised or semi-supervised labeling methods.

Furthermore, for small-scale applications we can identify some peculiarities or aspects that can be addressed differently from what happens in RS. Lighting, for instance, can be controlled and optimized: we think that the exploitation of broadband LED illumination sources in VNIR (400–1400 nm) and SWIR (1400–3000 nm) that are starting to appear on the market (https://www.metaphase-tech.com/hyperspectral-illumination/ (last visit March 2019)) can lead to a further expansion of applications, especially in the biomedical field or where power consumption and temperatures of the halogen lamps can be a problem. This is an interesting development perspective since HSI with LEDs has been often considered unfeasible.

Unlike RS, the problem of data transmission from satellites and the need for on-board compression is not present for small-scale applications. Still, the huge amount of collected data requires compression technologies as well, especially if there are medium- long-term data storage needs arising from statistical needs (e.g., in agricultural studies) or rather from exigencies related to food-traceability or medico-legal regulations. The relationship between compression effects and DL performance demands awareness, experimental validations and methods to guarantee the sought data quality. One pilot example in this direction can be considered the assessment of coding strength aimed at preserving DL-based classification performance in a biomedical application, as proposed in [109].

## 6. Conclusions

The richness of information contained in HSI data constitutes an indubitable appealing factor especially in sectors that benefit from computer assisted interpretation of visible and invisible (to the human eye) phenomena. However, industrial and professional HSI technologies are subject to cost-benefit evaluations which lead to the need for enabling factors to activate their deployment potentialities. In these years, machine learning technologies are rapidly extending their range and, boosted by the advent of Deep Learning, they are revolutionizing the world of digital data analysis. In this review, we tried to analyze what is currently happening with the meeting of HSI and DL technologies by adopting a multidisciplinary perspective and making our work accessible to both domain experts, machine learning scientists, and practitioners.

Although mitigated by the fact that pixel- and spectral-based analysis tasks can count on an order of thousands training samples for HSI volume, one of the main issues that emerged as an obstacle for quality scientific production is the limited number of publicly available datasets. More in general, the number and quality of acquired data in the various disciplines remains a central issue for the development of sound, effective and broad scope HSI-DL solutions. Rather, the exploration of different DL approaches for the RS field can stimulate efforts and investments in the provision of quality HSI datasets. Moreover, for other application fields where the penetration of HSI technologies is still way behind, the possibility to approach complex visual tasks by means of DL solutions can be seen as an enabling factor and a possible driver for a new era in the deployment of HSI technologies for a wide spectrum of small-scale applications in industry, biology and medicine, cultural heritage and other professional fields.

## Figures and Tables

**Table 1:** HSI-DL studies exploiting CNNs represented by target use (columns) and field—task (raws).

	Feature Extractor	Spectral or Spatial	Spectral–spatial
RS–Classification	[68,133,134,135,136,137,138]	[50,54,61,139,140,141]	[57,62,142,143,144,145,146,147,148,149,150,151,152,153,154,155,156,157,158,159,160,161,162,163,164]
RS–Data fusion	[90,91,92,94,95]		
RS–Detection	[67]		
RS–Image processing		[55,79]	
Biomedical	[97]	[102,103,107]	[58,100,113]
Food-agriculture		[123,127,128]	[121,126]

**Figure A1:**
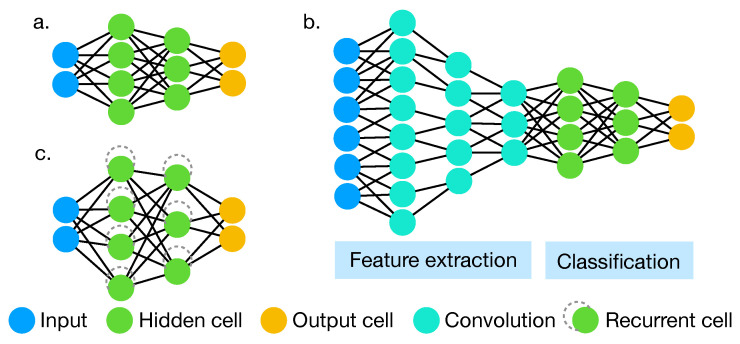
Network architectures. (**a**) Fully-connected; (**b**) Convolutional neural network; (**c**) Recurrent neural network.

**Figure A2:**
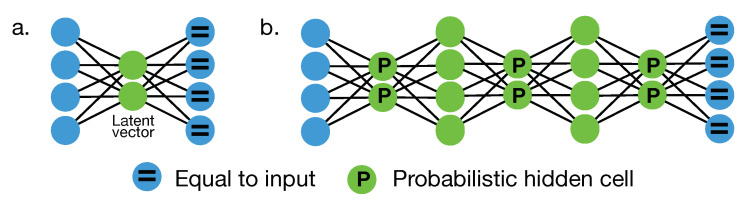
Network architectures. (**a**) Autoencoders; (**b**) Deep belief networks.

**Figure A3:**
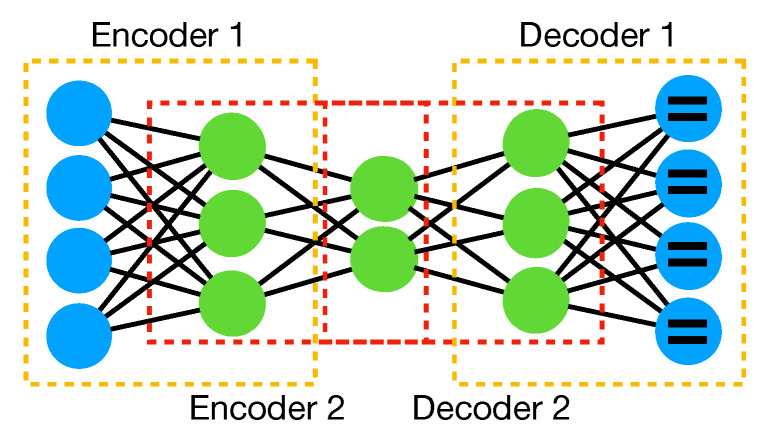
Network architecture of a Stacked Autoencoder

**Figure A4:**
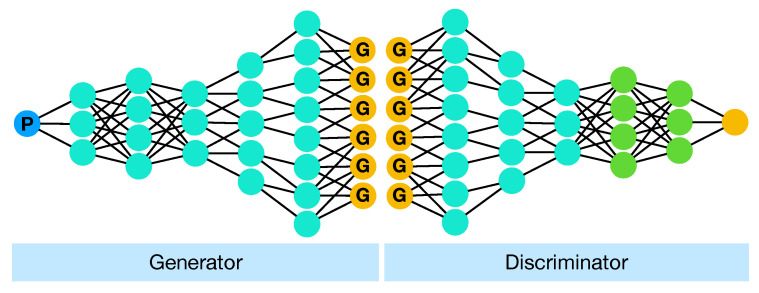
Architecture of Generative adversarial networks.

## Appendix A. DL Methods for HSI in Brief

Here, we give a brief introduction to the deep learning world to provide context and references to the core parts of this review. For a more extensive introduction to deep neural networks the reader can refer to [215], while the book [216] is a more comprehensive reference. In a RS perspective, valuable overviews of DL approaches can be found in [23,24,25].

DL is a branch of representational learning in which models are composed of multiple layers to learn representations from data in an end-to-end fashion. These methods have had a terrific impact to date and are expected to continue revolutionizing the way complex data analysis tasks are approached in domains such as natural language processing, speech recognition, visual object detection and recognition and many others. Together with the classical supervised and unsupervised learning approaches other paradigms have become relevant in the context of DL, where large amounts of data (order of hundreds of thousands) are supposed to be necessary to carry out correct learning of the high number of parameters characterizing a Deep model and to avoid overfitting. In fact, both sufficiently exhaustive data acquisition and labeling (supervision) can be costly or even unfeasible in some contexts. Different data augmentation strategies and techniques can be adopted and are common practice in many cases. Moreover, exploiting the fact that Deep architectures usually build a hierarchical bottom-up representation of the information, in many cases typically the lowest portion of a model trained on somehow related data in a source domain can be transferred to the target domain model, and so called transfer-learning approaches only require a residual estimation of a reduced portion of the parameters or allow a significant reduction of the learning epochs. Other ways to exploit knowledge, this time from the same target domain, belong to the wide family of semi-supervised learning methods. They allow to exploit the typical imbalanced presence of unlabeled data due to the difficulties, also characterizing many HSI domains, to produce large enough and high-quality labelled datasets. Semi-supervised learning can be operated for example by training a classifier with an additional penalty term coming from an Autoencoder (AE) or other unsupervised data embedding methods [217].

### Appendix A.1. Fully-Connected

When we refer to *fully-connected*, we are dealing with networks (or layers of a network) in which each node of a layer is connected to all the nodes in the following one without almost any constraints (see Figure A1a). Each neuron acts as a summation node with respect to its inputs. Eventually, a non-linear activation function is applied to the output. Fully-connected is one of the simplest layers and usually is used in the last part of the network for the final classification or regression.

### Appendix A.2. Convolutional Neural Networks

Convolutional neural networks (CNNs) [218] are particular types of deep feed-forward networks that are simpler to train, and more effective, on sampled data sources (Figure A1b). This is due to the constraints introduced in the hypothesis space that force a structure and reduce the number of parameters. The enforced structure creates features that are spatially invariant and robust to rotation and deformations (up to a certain amount). This is made possible thanks to local connections, shared weights and the use of pooling layers as well. CNNs are designed to process matrices or tensors such as colour images. Many data sources are in the form of multiple arrays: from 1D for sequences and signals, like audio or spectral signatures; 2D for images; and 3D for video or volumetric images.

Notable architectures are: AlexNet [219], which won the ImageNet competition in 2012, outperforming its competitor; GoogleLeNet [220], based on inception blocks which create sub-networks in the main network and increase either depth and width with respect to AlexNet; VGG [221] with its very small (3 × 3) and widely used convolution filter, and a simple and repetitive structure growing in depth; ResNet [222] that builds a very deep structure in which there are skip connections to let the information flow jump over a set or layers, solving the problem of vanishing gradients (i.e., the inability to propagate the error function backwards in very deep networks). This is because it has become too small after a certain point, and thus producing a potential stop of network training). If, instead, skip connections interconnect every following block, the architecture is called DenseNet [223]. Recently, many other networks focusing on low computational devices appear, such as MobileNet [224] and SqueezeNet [225], to name a few.

### Appendix A.3. Recurrent Neural Networks

Recurrent neural networks (RNNs) belong to an important branch of the DL family and are mainly designed to handle sequential data (see Figure A1c). A plain RNN is indeed not so powerful and seldom used in works nowadays. Rather, very high performance can be achieved with recurrent hidden units like LSTM (Long Short Term Memory) [226] or GRU (Gate Recurrent Unit) [227]. These units are composed of different internal data paths that can store and release information when needed and are capable of alleviating the vanishing gradient problem.

### Appendix A.4. Autoencoders

An autoencoder (AE) [228] is composed of: one visible layer of inputs, one hidden layer of units, one reconstruction layer of units, and an activation function (Figure A2a). During training, it first projects the input to the hidden layer and produces the latent vector. The network corresponding to this step is called the *encoder*. Then, the output of the encoder is mapped by a *decoder* to an output layer that has the same size as the input layer. The power of AEs lies in this form of training that is unsupervised and forces a meaningful compressed representation in its core. During reconstruction, AE only uses the information in hidden layer activity, which is encoded as features from the input. Stacking trained encoders (SAE, see Figure A3) is a way to minimize information loss while preserving abstract semantic information and improving the final model capacity.

### Appendix A.5. Deep Belief Networks

Deep Belief Networks (DBN) can be viewed as a composition of simple, unsupervised networks such as Restricted Boltzmann machines (RBM) [229] or autoencoders [230], in which each sub-network hidden layer serves as the visible layer for the next one (see Figure A2b). If necessary, a feed-forward network is appended for the fine-tune phase.

### Appendix A.6. Generative Adversarial Networks

Generative Adversarial Networks (GANs) have recently emerged as a promising approach to constructing and training generative models. In this framework there are two adversarial neural networks that are jointly trained: a *generator* *G* and a *discriminatorD* (see Figure A4). The generator is supposed to learn to generate the samples of a data distribution given random inputs, while *D* tries to discriminate between real data and artificially generated ones. The two networks are trained in a two-player minmax game scheme until the generated data are not distinguishable from the real ones. After a proper training procedure, *D* can be used as a well trained feature extractor, and applied to a specific problem with the addition of a final block that exploits the needed output (for instance a fully connected layer for classification).
